# Engineering an inducible gene expression system for *Bacillus subtilis* from a strong constitutive promoter and a theophylline-activated synthetic riboswitch

**DOI:** 10.1186/s12934-016-0599-z

**Published:** 2016-11-22

**Authors:** Wenjing Cui, Laichuang Han, Jintao Cheng, Zhongmei Liu, Li Zhou, Junling Guo, Zhemin Zhou

**Affiliations:** 1 School of Biotechnology, Jiangnan University, 1800 Lihu Road, Wuxi, 214122 Jiangsu China; 2 Key Laboratory of Industrial Biotechnology (Ministry of Education), Jiangnan University, 1800 Lihu Road, Wuxi, 214122 Jiangsu China

**Keywords:** Controllable gene expression, RNA switch, Theophylline, Synthetic riboswitches, *Bacillus subtilis*

## Abstract

**Background:**

Synthetic riboswitches have been increasingly used to control and tune gene expression in diverse organisms. Although a set of theophylline-responsive riboswitches have been developed for bacteria, fully functional expression elements mediated by synthetic riboswitches in *Bacillus subtilis* are rarely used because of the host-dependent compatibility between the promoters and riboswitches.

**Results:**

A novel genetic element composed of the promoter P43 and a theophylline-riboswitch was developed and characterized in *B. subtilis*. When combined with a P43 promoter (P43′-riboE1), the theophylline-riboswitch successfully switched the constitutive expression pattern of P43 to an induced pattern. The expression mediated by the novel element could be activated at the translational level by theophylline with a relatively high induction ratio. The induction ratios for P43′-riboE1 by 4-mM theophylline were elevated during the induction period. The level of induced expression was dependent on the theophylline dose. Correspondingly, the induction ratios gradually increased in parallel with the elevated dose of theophylline. Importantly, the induced expression level was higher than three other strong constitutive promoters including P_srfA_, P_aprE_, and the native P43. It was found that the distance between the SD sequence within the expression element and the start codon significantly influenced both the level of induced expression and the induction ratio. A 9-bp spacer was suitable for producing desirable expression level and induction ratio. Longer spacer reduced the activation efficiency. Importantly, the system successfully overexpressed β-glucuronidase at equal levels, and induction ratio was similar to that of GFP.

**Conclusion:**

The constructed theophylline-inducible gene expression system has broad compatibility and robustness, which has great potential in over-production of pharmaceutical and industrial proteins and utilization in building more complex gene circuits.

## Background

Controllable gene expression systems are important for determining the timing and strength of heterologous protein expression. The diversity and availability of easy-to-use biological parts enables the development of sophisticated devices based on tuneable gene expression systems. These devices and systems have been successfully utilized for the overproduction of recombinant proteins as well as for rewiring metabolic pathways [1]. A number of biological elements derived from natural operons, such as the IPTG-induced *lac* operon and the arabinose-activated *araC* promoter, have been widely used for conditional gene expression in *Escherichia coli* and *Bacillus subtilis*, two commonly utilized hosts in synthetic biology [2, 3]. However, there are still obvious drawbacks when employing these genetic tools in diverse hosts. For example, it has been broadly recognised that protein-based ligand-inducible tools that well functioned in *E. coli* do not work well in other bacteria, such as *B. subtilis*.

To overcome the limitations of protein-based gene expression tools, RNA-based elements have increasingly been viewed as superior genetic tools for a range of applications in synthetic biology [4, 5]. One such RNA regulator is the riboswitch, which is an RNA-encoded genetic control element that regulates gene expression in a ligand-dependent fashion without the need for proteins [6]. Riboswitches are composed of an aptamer domain, which recognizes the ligand, and an expression platform, whose conformational change is induced by the ligand binding to the aptamer, which directly modulates transcriptional termination or translational initiation [7, 8]. Ligand-inducible expression systems are essential genetic tools for commonly used bacteria such as *E. coli* and *B. subtilis*. Recent reports demonstrated an amino acid-activated riboswitch from *B. subtilis* that contains tandem glycine aptamers. Heterologous gene expression was activated by the glycine-riboswitch when it was bound to the added glycine [9]. Although the glycine-riboswitch system only resulted in a sixfold induction, it is still a useful model for the development of more riboswitch-dependent gene expression systems and other biological devices.

In addition to natural riboswitches that control gene expression in response to endogenous metabolites, a variety of synthetic riboswitches that respond to non-endogenous small molecules have been developed to control heterologous gene expression in different hosts [10, 11]. Using rational design, the Gallivan’s laboratory developed a set of synthetic riboswitches referred to as A-E that are activated by theophylline rather than the native metabolites. When combined with diverse synthetic ribosome binding sites (RBS), these artificial elements are able to function in various Gram-negative and Gram-positive bacteria to regulate translation [12]. Theophylline, which bears structural and pharmacological similarity to theobromine and caffeine, is a cheap and nontoxic chemical, enabling it to be an ideal inducer for triggering heterologous expression in microbial hosts [12]. The universality and availability of these synthetic riboswitches in other bacteria, such as *Mycobacteria*, *Streptomyces*, and *Cyanobacteria*, has been investigated. The performance of these riboswitches, including their activities and induction efficiency, varied according to the host organism, especially, when different promoters were placed upstream [13]. Therefore, the performance of a given riboswitch relies on different determinants, so no one riboswitch is well-suited to all genetic contexts.

*Bacillus subtilis*, a low G + C%-containing Gram-positive soil bacterium, has been commonly used as a host strain for producing numerous industrial proteins and fine chemicals because of several characteristics, including its status as generally recognized as safe (GRAS), its high cell density, its well-characterized protein secretion mechanism, the existence of well-established methods for its genetic manipulation, and its applicability for large-scale industrial production [14, 15]. Recent studies have concentrated on using *B. subtilis* to developed a sophisticated synthetic biology system, which depends on complete genome sequencing, reannotation, and the use of various biological tools for gene manipulation [16]. Although conditional control of gene expression by many protein-based elements in *B. subtilis* has been well-characterized and routinely used, the activation and tuning of gene expression with these elements depends on addition of expensive chemicals, which limit their scalability for industry application [1]. Recently, a set of synthetic theophylline-riboswitches was developed and optimized to function in diverse bacteria. Among these, the riboswitch E used with a constitutive promoter is reportedly suitable for *B. subtilis* [12]. However, one report showed that the gene expression level induced by the promoter-riboswitch combination did not consistently correlate with the promoter strength in certain bacteria [17]. This suggests that the compatibility of the riboswitch with specific homologous or heterologous promoters will be broad or narrow, respectively, in *B. subtilis* and other bacteria. The influence of combing riboswitch with other constitutive promoters on protein expression has not been well characterized until now.

To expand the toolbox for conditional gene expression in *B. subtilis*, a strong constitutive promoter, P43, was placed upstream of the synthetic riboswitch E1 (derived from the reported riboswitch E [12]). By using green fluorescent protein (GFP) as a reporter gene, riboswitch E1 with P43 was able to achieve inducible expression in *B. subtilis*. The conditional expression pattern was theophylline-dependent with high stringency over a wide range of concentrations. Moreover, the optimal spacing between the SD sequence and the start codon within the synthetic element was also determined by varying the distance. This resulted in the successful engineering a strong constitutive promoter with an RNA regulator-based element, which could be broadly applied in controlling heterologous gene expression and used as an element for input signal in construction of complex gene circuits.

## Results

### The strong constitutive P43 promoter was successfully combined with a theophylline-activated expression element

**Fig. 1:**
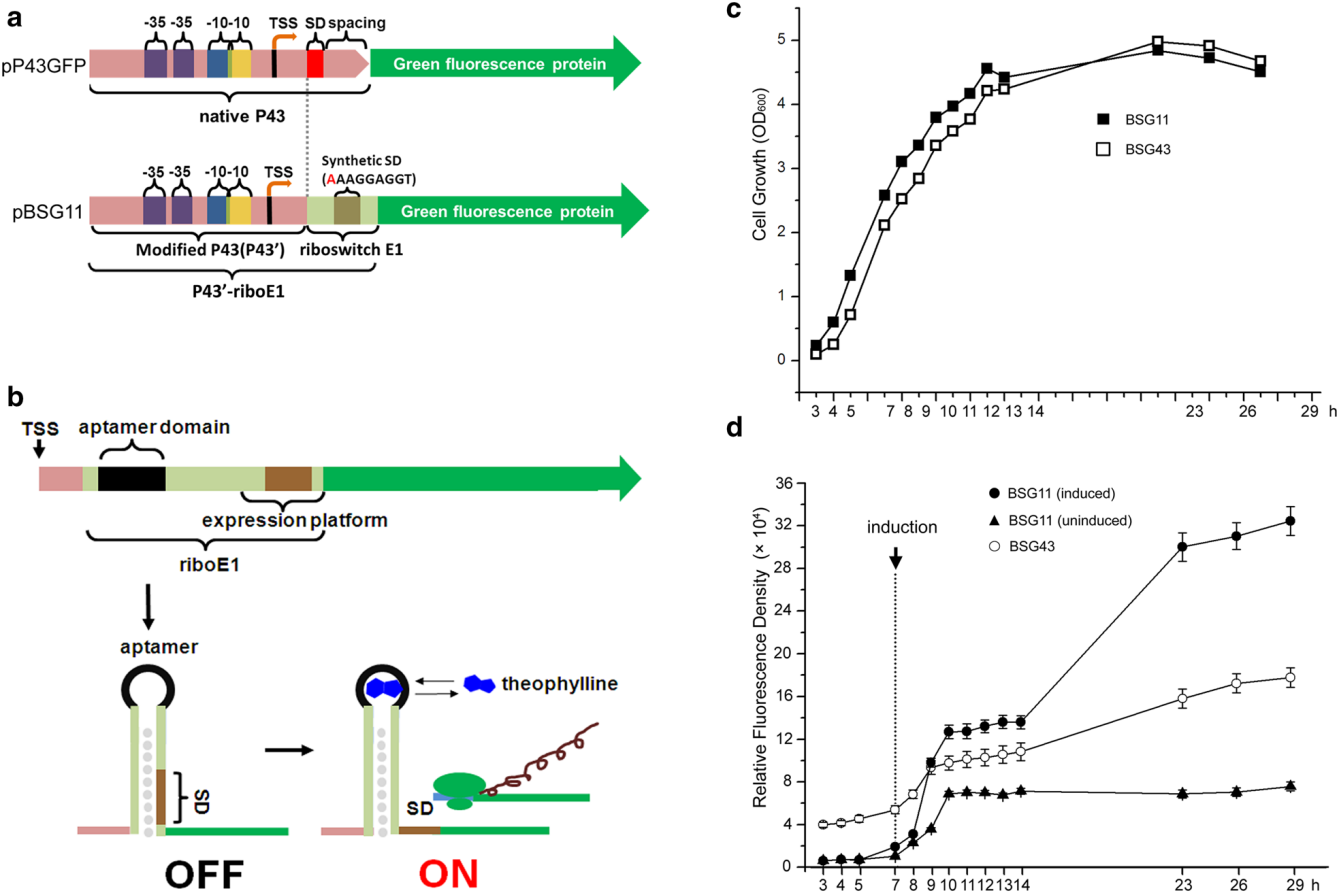
The combination of the novel theophylline-dependent riboswitch E1 and the P43 promoter in *Bacillus subtilis*. **a** Diagrams of the expression cassette driven by the native P43 promoter and the novel theophylline-dependent expression element (P43′-riboE1). The* green* fluorescent protein is represented by the *green arrows*, and the transcription start site is marked as TSS. The promoter fragment of the novel expression element, P43′, was produced by deletion of the entire sequence downstream of the native P43. The synthetic riboswitch E1 was placed immediately downstream of P43′, resulting in the novel expression element P43′-riboE1. The riboswitch was derived from the riboswitch E (previously reported by Topp et al.), in which a single A was inserted before the 5′ terminus of the original sequence. In the diagram, the inserted nucleotide is coloured *red*. The *dash line* denotes the difference between native P43 and the synthetic riboswitch E1 (riboE1). **b** The schematic diagram of mechanism of the novel genetic element. Theophylline is indicted in *blue*. **c** The growth curves of *B. subtilis* 168 harbouring pP43-*gfp* (BSG43) and pBSG11 BSG11). The recombinant *B. subtilis* strains were inoculated by pre-cultures with the initial OD_600_ of 0.05. The cultures were sampled periodically to measure the cell density until the cell density began to decrease at 27 h. **d** Expression levels of GFP (*y*-axis) against time (*x*-axis) during the culture period. The *dashed line* denotes the induction by 4 mM theophylline. The GFP fluorescence was measured in triplicates and the data were shown in mean ± SD

Previously many diverse theophylline-dependent riboswitches were engineered and shown to function in various Gram-positive and Gram-negative bacteria at the translational level [12]. However, it was unclear if these riboswitches were compatible with most constitutive promoters. Usually, constitutive promoters are paired with theophylline riboswitches to achieve controllable and tuneable transcription [17, 18]. Here, a new riboswitch, theophylline riboswitch riboE1 containing a modified SD sequence AAAGGAGG was constructed by modifying the riboswitch E constructed by Topp et al. [12]. RiboE1 was genetically fused to a strong promoter P43, in which the native downstream SD sequence was deficient (P43′), yielding a novel dual expression element P43′-riboE1. The P43′ retains the full upstream sequence including the core region (−35 and −10), the transcriptional start site (TSS), and the 5′UTR between the TSS and the native SD sequence. The synthetic theophylline riboswitch is composed of an aptamer region, a synthetic SD sequence, and a spacer (Fig. 1a). Theoretically, the transcription of the riboE1 control element is triggered by P43 during the cell growth. However, the synthetic SD located downstream of the aptamer is sequestered via pairing with the bases in the stem of the riboswitch, resulting in translational block. The binding of theophylline to the aptamer domain initiates translation by altering the downstream base pairing, which releases the SD. Subsequently, GFP expression begins after the ribosome binding to the SD (Fig. 1b). We compared the cell growth and the expression pattern of GFP between the constitutive expression system driven by P43 and the inducible expression system driven by P43′-riboE1. The growth curve showed that the increase in cell density was similar for both the recombinant strain BSG11 (*B. subtilis* harbouring pBSG11, inducible expression of GFP) and the BSG43 (*B. subtilis* harbouring pP43-*gfp,* constitutive expression of GFP) strain during the culture period (Fig. 1c). This indicates that the recombinant strains BSG11 and BSG43 grow identically.

Meanwhile, the constitutive expression in BSG43 carrying pP43-*gfp* and the inducible expression in BSG11 carrying pBSG11 were monitored by measuring the GFP fluorescence intensity in the cultures. The data showed that GFP fluorescence intensity for BSG43 increased gradually during the culture period. After 29 h, the relative fluorescence units reached approximately 180,000 (a.u/OD_600_). In contrast, the GFP fluorescence in BSG11 displayed very low levels in the absence of theophylline. After culturing for 7 h, 4 mM theophylline was added to the medium, and GFP fluorescence dramatically increased until peaking at approximately 320,000 (a.u/OD_600_), whereas the BSG11 without induction kept constant low leaky expression levels over the cultural process (Fig. 1d). This indicated that the fusion of the theophylline-dependent riboswitch to the P43 resulted in a controllable expression element.

### Characterization of the induction levels and ratios driven by P43′–riboE1 element

**Fig. 2:**
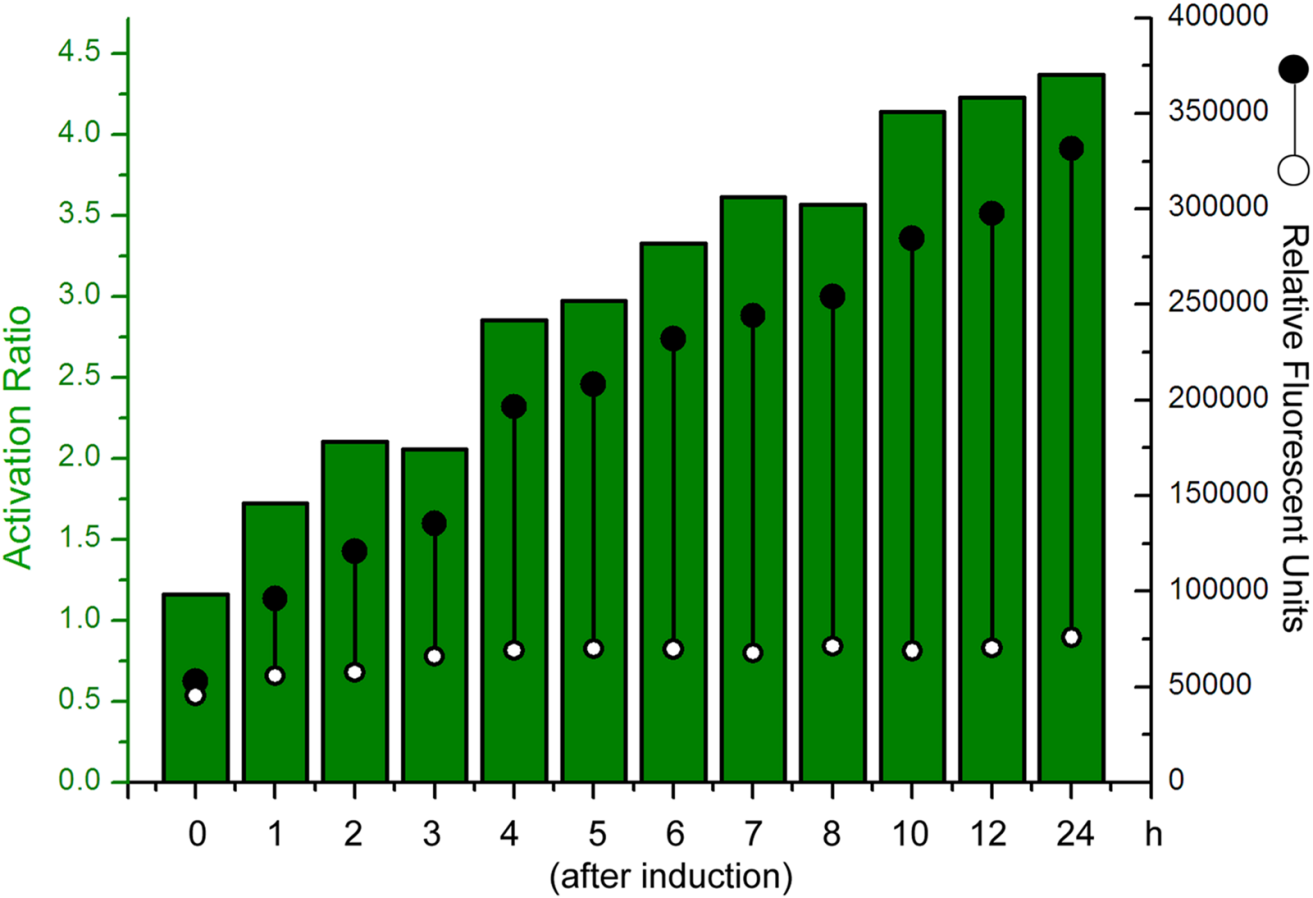
Characterization of induction of GFP from pBSG11 harbouring P43′-riboE1 element after induction by 4-mM theophylline. GFP induction is denoted by* green* histograms at different time points. The *solid circles* within in the histograms represent the level of induced GFP expression. Similarly, the *open circles* within the histograms are the corresponding basal expression level. The activation ratio was obtained by dividing the induced expression level by the corresponding basal level

To characterize the activated level and efficiency driven by the P43′-riboE1 element, the BSG11 strain was cultivated in LB medium for 7 h prior to treatment with 4 mM theophylline. Figure 2 shows the theophylline-dependent induction of GFP fluorescence after culturing for 24 h. The histograms show that the activated expression levels of GFP and the induction ratios increased gradually during the induction period. After 24 h of induction, the expression level exceeded 350,000. The induction ratios constantly increased during the induction period, and were consistent with the expression levels. Expression due to leakage remained low during the induction period. Finally, the induction ratio peaked at 4.3 after 24 h of induction (Fig. 2).

### Theophylline-activated gene expression by P43′-riboE1 was dose-dependenct

**Fig. 3:**
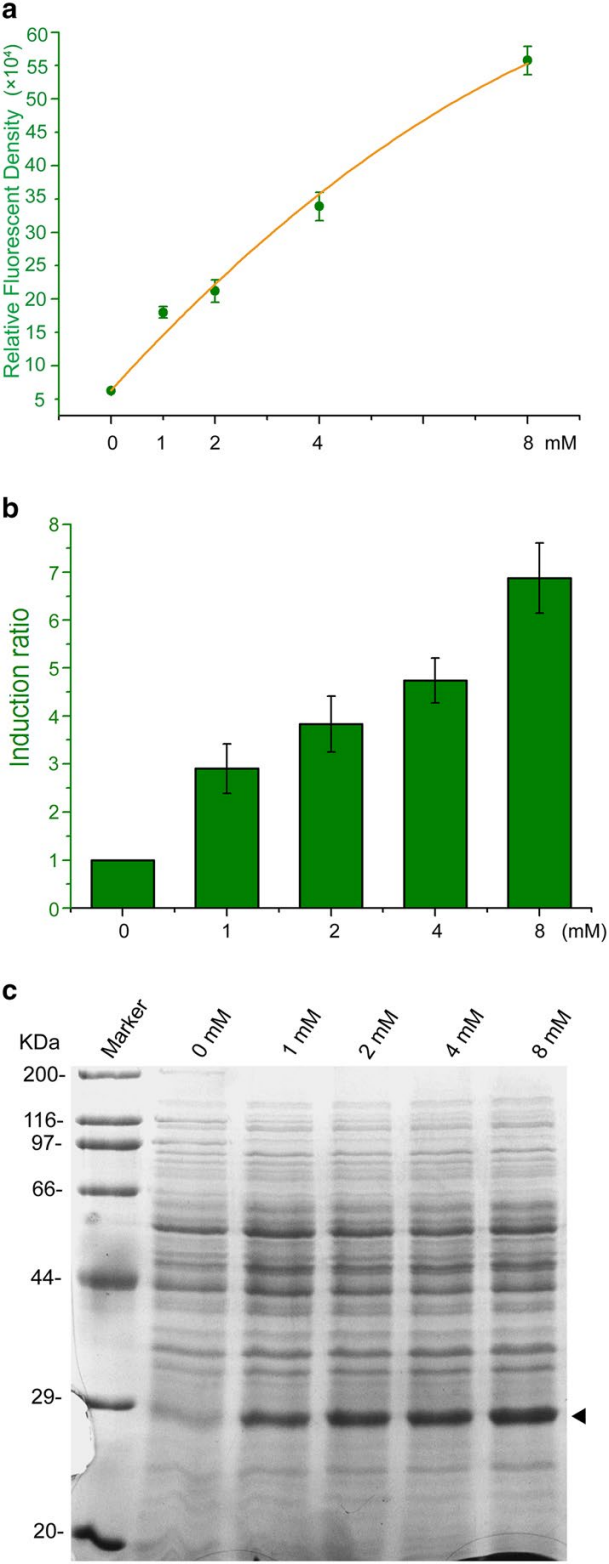
The activation of GFP expression by the P43′-riboE1 element displayed dose-dependency. **a** The expression levels of GFP induced by 1, 2, 4, or 8 mM theophylline were determined 24 h after induction, and were linearly correlated. The experiment was repeated independently in triplicates. **b** The activation ratios were estimated after induction of 1, 2, 4, or 8 mM theophylline. The ratios were elevated in response to the increased theophylline levels. The highest activation ratio was achieved by 8 mM theophylline, which caused an approximately sevenfolds increase. **c** SDS-PAGE analysis of with increasing amounts of theophylline. The *black solid arrow* indicates the GFP band. Twenty micrograms of total protein were loaded for each sample. The GFP fluorescence was measured in triplicates and the data were shown in mean ± SD

To examine whether the activation of GFP by theophylline was dose-dependent, the cultivated BSG11 strain was treated with 4% DMSO (0 mM theophylline) or 1, 2, 4 or 8 mM theophylline for 24 h. Then, the fluorescence was measured to determine the dose-dependency. The GFP expression level increased linearly from 0 to 4 mM and continued to increase for the higher concentration of theophylline (up to 8 mM) (Fig. 3a). In particular, the induction ratios were dramatically elevated with the increasing doses of theophylline and reached 6.8 at 8 mM theophylline dose (Fig. 3b). These results indicated that the higher doses of the inducer elicited higher expression levels and a higher induction ratio. Moreover, the overall expression levels of GFP showed dose-dependence under the control of this riboswitch element. Afterwards, SDS-PAGE analysis was used to examine GFP expression under different concentrations of theophylline. The results showed that the accumulation of GFP within the bacterial cells increased at higher theophylline concentrations, which was consistent with the fluorescence assays (Fig. 3c). These results indicated that the expression level of genes mediated by the P43′-riboE1 element can be controlled with different concentrations of the inducer.

### Comparison of theophylline riboswitch-controlled gene expression levels with expression driven by other constitutive promoters

**Fig. 4:**
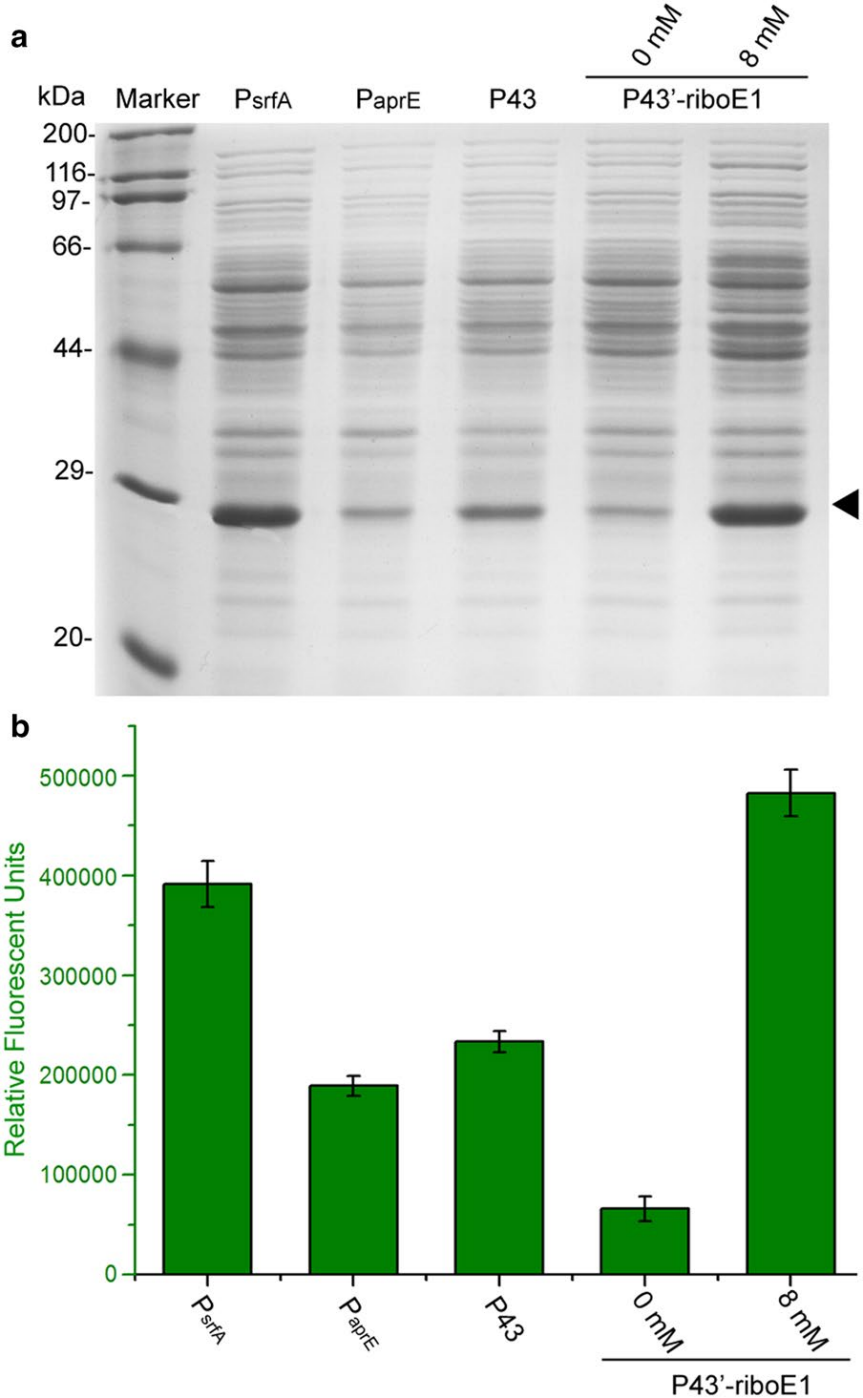
Comparison of the induced expression level with P43′-riboE1 and three strong promoters from *B. subtilis*. **a** SDS-PAGE analysis of GFP controlled by the constitutive promoters P_srfA_, P_aprE_, P43 and by the theophylline-induced element P43′-riboE1. The BSG11 strain was activated by 8-mM theophylline for 24 h prior to sampling for SDS-PAGE. **b** Fluorescence intensity representing the relative expression level was driven by three constitutive promoters as well as by the P43′-riboE1 element after induction by 8-mM theophylline for total 31 and 24-h culture periods, respectively. The GFP fluorescence was measured in triplicates and the data were shown in mean ± SD

To comprehensively compare the expression levels between commonly used strong constitutive promoters and P43′-riboE1, three wild-type promoters P43, P_aprE_ and P_srfA_, which have been characterized in previous studies [19, 20], were employed to express the GFP reporter. BSG43, BSG04, and BSG03 carrying the three strong promoters respectively, were cultured in LB for 31 h. The BSG11 harbouring pBSG11 was treated with 8 mM theophylline for 24 h. Then, the final expression level was determined by SDS-PAGE analysis. The GFP expression level controlled by P43′-riboE1 was far higher than expression by P_aprE_ and P43 in the presence of 8-mM theophylline and was equivalent to expression by P_srfA_. The basal level of P43′-riboE1 was relatively low, which was consistent with the fluorescence intensity shown in Fig. 2. Interestingly, the final expression level mediated by P43′-riboE1 in the presence of 8 mM theophylline was somewhat higher than expression by P43 (Fig. 4a). To quantitatively determine the differences in the expression levels controlled by the four types of gene expression elements, fluorescence intensity was measured. The fluorescence of GFP driven by wild-type promoters and the activated riboswitch element showed a trend similar to the SDS-PAGE analysis of GFP expression (Fig. 4b). This demonstrated that highly controllable and tuneable gene expression can be achieved by using P43′-riboE1 element.

### A 15-bp spacer between the start codon and the riboswitch element affects induction

**Fig. 5:**
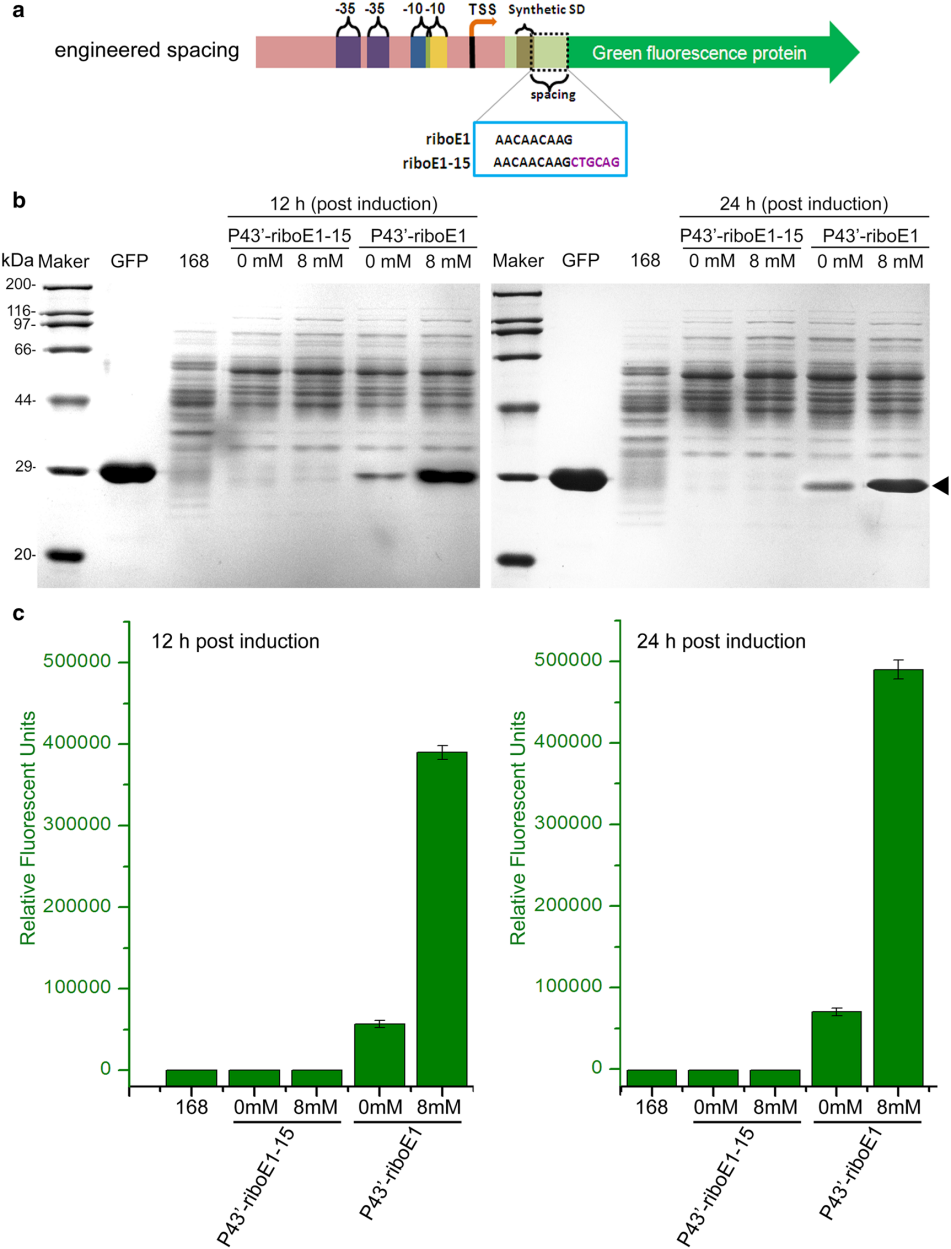
Determination of the effect of length of spacer on the induced level of GFP expression. **a** Schematic diagram displays the gene structure of riboE1, and riboE1-15 harbouring 15-bp spacer between SD sequence and start codon was produced by insertion of *Pst*I restriction site immediately downstream of the SD sequence. The SD sequences within riboE1 and the modified riboE1-15 are in the azure box. **b** SDS-PAGE analysis was carried out to detect the expression level of GFP driven by P43′-riboE1 and P43′-riboE1-15 after induction with 8 mM theophylline for both 12 and 24 h. The strains harbouring recombinant plasmids treated with 4% DMSO are designated as 0 mM (controls). Protein extracts from *Bacillus subtilis* 168, which were collected at 19 and 31-h culture (equal to the induced groups with 12 and 24-h induction, respectively), are used as the negative controls. The GFP stands for the purified GFP protein, which is served as the specific marker to indicate the corresponding position of heterologous GFP. **c** Measurement of the relative fluorescent units of GFP corresponding to the SDS-PAGE in **b**

To facilitate the cloning of the P43′-riboE1 element into other vectors, it was essential to modularize the element. One method is to insert restriction site either up- or down-stream of the element. Here, we inserted a *Pst*I restriction site immediately downstream of the P43′-riboE1, to produce pBSG10 that contains P43′-riboE1-15 with a 15-bp spacer (Fig. 5a). To compare the effect of the longer spacer on GFP expression, BSG11 and BSG10 were treated with 8 mM theophylline for 12 and 24 h, and the production of GFP was analysed by SDS-PAGE. Obviously, the GFP protein expression was scarcely detected in BSG10 cultures treated with 8 mM theophylline or DMSO treatments in both 12- and 24-h cultures (Fig. 5b). In contrast, GFP expression in BSG11 for 12- and 24-h cultures was substantially higher than in BSG10. Noticeably, the basal expression level in BSG11 also was significantly higher than in BSG10 for both 12- and 24-h cultures (Fig. 5b). Additionally, the GFP fluorescence in BSG10 and BSG11 corresponding to the SDS-PAGE was determined to quantify the expression level. The data were consistent with that measured by SDS-PAGE, which authenticated the difference in heterologous expression level between the two different spacers (Fig. 5c). These results suggested that the failure of P43′-riboE1-15 to induce GFP expression after exposure to theophylline was due to increased spacer sequence length between the SD and the start codon, which probably affects ribosome binding in the translation initiation step [21].

### Validation of the novel theophylline-responsive riboswitch element with β-glucuronidase

**Fig. 6:**
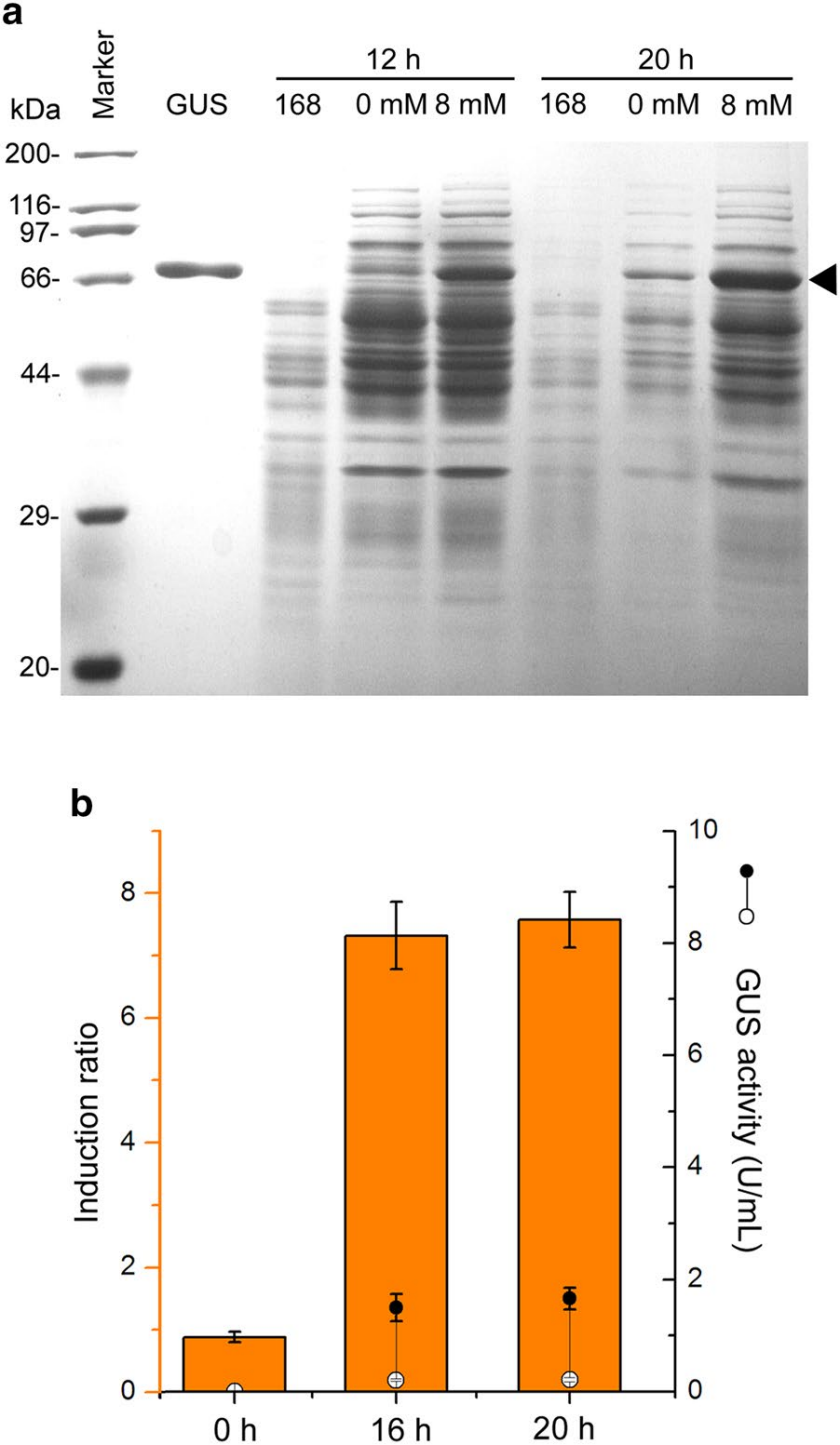
Validation of the theophylline-responsive gene expression system with *gus* reporter gene. **a** SDS-PAGE analysis showed the expression level of GUS in the absence and presence of theophylline. The induced expression level of GUS in the recombinant strain, BSGgus, was determined by treatment with 8 mM theophylline for 20 h culture, and the culture treated with 4% DMSO for the same time after induction was designated as controls (0 mM). The *solid triangle* pointed the overproduced GUS. Protein extracts from *B. subtilis* 168 sampled simultaneously with the induced recombinant strains was used to be the negative control. GUS, denoted the purified GUS protein, was used to be a specific marker to indicate the bands of GUS on SDS-PAGE. **b** Enzymatic assay for GUS in BSGgus after induction by 8 mM theophylline for 16 and 20 h. The induced activities of GUS and the levels for leakage are shown by *solid* and *open circles*, respectively. The histograms represent the corresponding induction ratios. The enzymatic activity assay was performed in triplicates and the data are presented in mean ± SD

To test the usefulness and the compatibility of the gene system, the reporter gene *gus* was used to verify the expression level driven by P43′-riboE1 element. The SDS-PAGE analysis showed some level of *gus* expression in both the presence and absence of theophylline. The *gus* was successfully activated at very high level after treatment with 8 mM theophylline for 16 and 20 h. In contrast, the control groups treated with 4% DMSO for the same time showed relatively low GUS expression levels (Fig. 6a).

The enzymatic activities and induction ratio of GUS were determined by measuring the GUS activity. The induction ratio in 16- and 20-h induced cultures was 7.3 ± 0.4 and 7.6 ± 0.4 U/mL, and the activated activity of GUS was 1.5 ± 0.2 and 1.7 ± 0.2 U/mL, respectively. Meanwhile, levels for both GUS activity from expression leakage at 16- and 20-h were 0.2 ± 0.03 and 0.3 ± 0.03 U/mL (Fig. 6b), which were rather low compared to that with induction. Approximately equal expression levels in 7-h cultures before the addition of theophylline were observed between the two experimental groups. These results were consistent with those for GFP expression, indicating that the controlled expression system constructed by combining a constitutive promoter and the theophylline-responsive riboswitch, potentially has broad compatibility with numerous heterologous genes and is widely applicable in synthetic biology.

## Discussion

Here, a novel controllable expression element composed of an engineered theophylline-dependent riboswitch and the strong constitutive P43 promoter was successfully constructed and characterized in *B. subtilis*, and inducible heterologous gene expression was efficiently achieved. Compared to the commonly used inducer, IPTG, theophylline is cheaper and nontoxic, which has potential utilization in triggering heterologous gene expression in microbial hosts. The riboswitch E is one of six riboswitches activated by theophylline that has the potential to induce gene expression, but, it has a low induced expression level in *B. subtilis* [12].

In the Gallivan’s study on fabrication of diverse theophylline riboswitch A–E*, we noticed that the difference in the synthetic SD sequence influenced the induction features in diverse bacteria [12]. Thus, to improve the level of expression in this study, a single adenine base was inserted upstream of the native SD sequence to generate a SD sequence with full complementarity to the ribosome. The highest induction ratio for this novel element was 6.8 after treatment with 8 mM theophylline (Fig. 3b), which was lower than that combined with other promoters in *Cyanobacteria* [13] and *B. subtilis* [12]. These unexpected results obviously indicate that the novel riboswitch-mediated element produced higher expression than previously reported, but at the cost of a decreased induction ratio. Riboswitches combined with different promoters display distinct properties in various bacteria [12, 18, 22, 23]. Therefore, it is worthwhile to assess the performance of riboswitch elements in the context of individual genetic backgrounds. Usually, several features such as regulatory efficiency, basal activity, noise, and kinetic parameters are systematically considered under similar experimental settings [24]. The continuous increase in the induction ratios over activation at different times suggested that the induction by P43′-riboE1 functions regularly in the *B. subtilis* host.

Although it has been shown that the riboswitch A, E* and the riboswitch F derived from the E combined with different promoters are able to function in *Streptomyces* [25], *Cyanobacteria* [13], and *Mycobacteria* [18], which displayed both high induction ratios and high expression levels, it is still unclear whether a single riboswitch is suitable for fabricating expression elements with diverse constitutive promoters that have a consistent induction ratio and similar dynamics. It is difficult to predict the activation characteristics of specific riboswitch-promoter combinations merely from induction data from the same riboswitch fused to other promoters. As the synthetic theophylline riboswitches function by controlling accessibility to the RBS [12, 26], promoters positioned upstream of the theophylline riboswitch-controlling elements, which share the same functional regions with the corresponding wild-type promoters, ought to output the similar rate of transcription to the corresponding wild-type promoters, and result in comparable translational levels. However, the final expression level of GFP in the P43′-riboE1-harbouring strain (BSG11) was higher than that in the P43-harbouring strain (BSG43) (Fig. 4a). Based on a recent report that described the effect of upstream secondary structure within the 5′UTR on translation efficiency [27], we proposed that the aptamer domain of the theophylline riboswitch naturally forming a stem-loop upstream of the SD sequence after transcription augments the accessibility of the ribosome to the SD, eventually producing higher levels of GFP. These results are consistent with previously reported data in which the induced expression levels altered after combining different promoters with the same riboswitch [22, 23].

The capability to control and tune the expression of proteins is an advantageous feature of synthetic riboswitches. Some theophylline riboswitches have been further improved by their combination with different promoters to achieve dose-dependent induced expression levels, allowing for the precise regulation of a gene [13, 28]. The theophylline riboswitch combined with the P43 promoter that was constructed in this study can be induced to different degree across a wide range of concentrations and at the same time produce corresponding induction ratios (Fig. 3). This gives the P43′-riboE1 broad utility for regulating gene expression. Although the P43′-riboE1 expression element produced lower induction ratios than theophylline riboswitch in other bacteria at same concentrations of theophylline, it produced higher levels of expression. In several Cyanobacterial species, theophylline riboswitches combined with native strong promoter ConII led to high induction ratios mediated that were achieved at 2 or 3 mM inducer concentrations [13]. Even in other Gram-positive bacteria, such as *Mycobacteria* and *Streptomyces*, high levels of induced gene expression, controlled by riboswitch E* fused to other constitutive promoters, were obtained with treatment of no more than 5 mM theophylline [18, 22, 25].

The length of the spacer between the SD sequence and the start codon has been shown to strongly influence the efficiency of translation initiation [21]. In the Gram-negative bacteria *E. coli*, only rare mRNAs have spacer lengths shorter than 7 bp and longer than 15 bp. The optimal length is approximately 9 bp [27]. Although the principle is derived from a systematic analysis of *E. coli* native genes, it might also be suitable to synthetic genetic elements such as riboswitches. A 15 bp length between the SD sequence and the start codon strikingly impaired the expression of GFP, indicating that a longer spacer length decreases the translation initiation efficiency (Fig. 5b). The optimal spacing length occurring in the native mRNAs is reported to be approximately 10 bp long, which improves the efficiency of translation initiation. However, in this study, the desired expression level and activation efficiency were obtained with 9-bp spacer. This inconsistency suggests that the gene expression controlled by a synthetic riboswitch functions in a more complex way in different bacteria. The range of use for this novel expression element was verified by production of the commonly used reporter, *gus*. The highest induced level and induction ratio of GUS in this study was 1.6 and 7.5 U/mL, respectively. The induction ratio was similar to that calculated for GFP. The leaky expression level corresponding to the highest expression level was 0.3 U/mL with this element, which was significantly lower than that with induction. Lower leaky expression level produced by riboswitch E was also observed in other theophylline-mediated inducible system in *Streptomyces coelicolor*, in which the leakiness of GUS level is 0.1 GU. However, the induced expression level of GUS in the *Streptomyces* system was lower than that in our construct [22].

## Conclusion

In this work, we constructed an inducible gene expression element composed of theophylline-dependent riboswitch and a constitutive promoter and characterized the induction properties in *B. subtilis*. Our data demonstrated that the P43 has good compatibility to the engineered riboswitch, which displayed high activation ratio and expression level in a dose-dependent manner. Combination of P43 to the engineered theophylline riboswitch did not decrease the expression but enhanced the expression level. Two reporters, GFP and GUS, were successfully over-expressed in recombinant *B. subtilis* by activation with theophylline. These results imply that the element reported here was efficient at tuning gene expression to a wide range of levels in *B. subtilis*. In summary, the novel theophylline-dependent riboswitch fused with strong constitutive promoter is able to efficiently drive gene expression in *B. subtilis*. The properties of high induction efficiency and controllable expression level are not only of great interest for constructing and designing more complex genetic circuits in *B. subtilis* but they also provide a more diverse set of genetic tools for synthetic biology.

## Methods

### Strains and culture conditions

The bacterial strains and plasmids that were used in this study are listed in Table 1. *Escherichia coli* JM109 was used for cloning and plasmid propagation, and *B. subtilis* 168 and its derivatives were used for evaluation of the inducible riboswitch. All of the strains were cultured with aeration in Luria–Bertani (LB) at 37 °C. When appropriate, the *B. subtilis* growth medium was supplemented with kanamycin (5 μg/mL), and the *E. coli* growth medium was supplemented with ampicillin (10 μg/mL). Cell density was determined by measuring the OD_600_ with a UV-1800/PC spectrophotometer (MAPADA Instrument Co., Ltd., Shanghai, China). The plasmid pBSG03 was used as a backbone to construct the plasmids containing various riboswitch elements. Plasmid pUC19A harbouring the synthetic riboswitch E1 (hereafter referred to as riboE1) was used as the cloning template and was provided by the Shanghai Sangon Biotech Co., Ltd. (Shanghai, China).

**Table 1:** Strains and plasmids used in this study

Plasmids	Properties and characteristics	Reference
pBSG03	P_srfA_, *gfp*, *bla*, *Km* ^r^, shuttle vector	[20]
pBSG26	P_aprE-HpaII_, promoter, BS*AP(aminopeptidase)*, *bla*, *Km* ^r^, template for *aprE* Promoter (P_aprE_)	[19]
pUC57B	pUC19 derivative, P_aprE_ fused with modified theophylline riboswitch E (riboE1, 9-bp spacer), *bla*	This study
pP43-*gfp*	pBSG03 derivative, ΔP_srfA_::P43(from *Bacillus subtilis* 168), *gfp*, *bla*, *Km* ^r^	This study
pBSG04	pBSG03 derivative, ΔP_srfA_::P_aprE_, *gfp*, *bla*, *Km* ^r^	This study
pBSG61	pBSG03 derivative, ΔP_srfA_::P_aprE_-riboE1	This study
pBSG11	pBSG61 derivative, ΔP_aprE_::P43, riboE1, *gfp*, *bla*, *Km* ^r^	This study
pBSG10	pBSG11 derivative, insertion of *Pst*I endonuclease immediately downstream of the 9-bp spacer (15-bp spacer), riboE1-15, *gfp*, *bla*, *Km* ^r^	This study
p43E-*gus*	pBSG11 derivative, Δ*gfp*::*gus*, riboE1, *bla*, *Km* ^r^	This study
Strains		
*E. coli* JM109		Lab stock
*B. subtilis* 168	trpC2	Lab stock
BSG11	*B. subtilis* 168 derivative, harbouring pBSG11	This study
BSG10	*B. subtilis* 168 derivative, harbouring pBSG10	This study
BSG61	*B. subtilis* 168 derivative, harbouring pBSG61	This study
BSG04	*B. subtilis* 168 derivative, harbouring pBSG04	This study
BSG43	*B. subtilis* 168 derivative, harbouring pP43-*gfp*	This study
BSGgus	*B. subtilis 168 derivative*, harbouring p43E-*gus*	This study

### Gene manipulation

*Escherichia coli* JM109 was used for propagating all plasmids. Inverse-PCR was used to insert or substitute target genes into the plasmid as previously described [20]. *Dpn*I (New England BioLabs) was used to digest the template plasmid after the inverse-PCR reactions. PrimeSTAR^®^ DNA Polymerase (TaKaRa Bio Company, Dalian, China) was used for the PCR reactions. A DNA extraction kit was purchased from TIANGEN Biotech (Beijing) Co., Ltd. (Beijing, China) and was used for plasmid DNA isolation.

### Plasmids construction

All of the plasmids used in this study are listed in Table 1. All the primers used in this study were shown in Table 2. The synthetic theophylline riboswitch E [12] (riboE for short) combined with the truncated *aprE* promoter lacking the downstream sequence of the native SD (termed P_aprE’_), was previously synthesized and then cloned into pUC19 by Shanghai Sangon Biotech Co., Ltd, the resulting plasmid was called pUC57A. pUC57B, carrying the novel riboswitch element riboE1 (Table 3) with a modified SD sequence containing a single A upstream of the SD sequence from pUC57A, was constructed by whole-plasmid inverse PCR with primers p57F and p57R and used as the template to build the following *E. coli*–*B. subtilis* shuttle vector containing the theophylline riboE1. To sub-clone the riboE1-mediated expression element into the *E. coli*–*B. subtilis* shuttle vector, the P_aprE’_-riboE1 fragment and 36-bp flanking sequences with homology to the upstream and downstream regions of *srfA* promoter (P_srfA_) on plasmid pBSG03 were amplified using primers G61F and G61R. This fragment was used as a mega-primer for whole-plasmid inverse PCR to insert the template into plasmid pBSG03 in place of P_srfA_ [19], yielding shuttle vector pBSG61. The expression level from the native *aprE* promoter in pBSG04 was compared to expression from P_srfA_ and P43. The pBSG04 plasmid was constructed in two steps. First, the native P_aprE_ was amplified from pBSG26 using primers G04F and G04R, resulting in a 573-bp fragment and 36-bp flanking sequences homologous to the up- and down-stream sequences of P_srfA_ from pBSG03. The fragment was inserted into pBSG03 using aforementioned inverse-PCR method. This resulted in the substitution of the P_srfA_ with P_aprE_ and yielding pBSG04. Similarly, the plasmid pBSG11 harbouring the P43 promoter combined with riboE1 was constructed in two steps. In the first step the P43′ (lacking the downstream SD sequence) and 36-bp flanking sequences homologous to P_aprE’_ from pBSG61 were amplified. Next, inverse PCR was used to substitute the P_aprE’_ upstream of the riboE1 with the P43 fragment. Plasmid pBSG10, which contains a longer (15-bp) spacer between the synthetic SD and the start codon relative to pBSG11 (Table 3), was constructed by inserting of a *Pst*I restriction site upstream of the start codon in pBSG11 by inverse PCR with the G10F and G10R primers. To compare the induced systems with constitutive system, pP43-*gfp* was constructed by substituting the P_srfA_ on pBSG03 by two steps. Firstly, native P43 from *Bacillus subtilis* 168 flanking 20-bp homologous sequence up- and down-stream of P_srfA_ on pBSG03 was amplified with primers pP43F and pP43R. Next, P_srfA_ on pBSG03 was replaced by P43 by inverse PCR using the purified amplified product in first step. To test the ability of the riboswitch element P43′-riboE1 to regulate the production of target proteins, the gene for the β-glucuronidase (*GUS,* EC: 3.2.1.31) reporter from *E. coli* JM109 was amplified using the primers E-gus F and E-gus R that contained the flanking sequences homologous to the upstream and downstream of *gfp* sequences in plasmid pBSG11. Then, the PCR product was purified and was used to replace the GFP sequence by inverse PCR, to produce p43E-*gus*.

**Table 2:** Primers used in this study

Primer name	Sequence (5′–3′)
G61F	**CAAAACCCCCCTTTGCTGAGGTGGCAGAGGGCAGGT**TGCCGAATTCCATGAACGAGACTTAAAA^a^
G61R	**GACAACTCCAGTGAAAAGTTCTTCTCCTTTACTCAT**CTGAGCAGGGTGCTTGTTGTTACCTCCTT^a^
G04F	**CAAAACCCCCCTTTGCTGAGGTGGCAGAGGGCAGGT**TGCCGAATTCCATGAACGAGACTTA^a^
G04R	**GACAACTCCAGTGAAAAGTTCTTCTCCTTTACTCAT**TCGGTTCCCTCCTCATTTTTATACC^a^
G10F	CCCTGCTAAAGGAGGTAACAACAAG*CTGCAG*ATGAGTAAAGGAGAAGAACTTTTCA^b^
G10R	TGAAAAGTTCTTCTCCTTTACTCAT*CTGCAG*CTTGTTGTTACCTCCTTTAGCAGGG^b^
E-gus F	CCCTGCTAAAGGAGGTAACAACAAGATGTTACGTCCTGTAGAAACCCCAACCCG
E-gus R	CAGATGCGTAAGGAGAAAATACCGCCTACCGGCCGCATAGGCCTTGTTTGCCTCCCTGCT
p57F	TGGCAGCACCCTGCTAAAGGAGGTAACAACAAGATG
p57R	CATCTTGTTGTTACCTCCTTTAGCAGGGTGCTGCCA
pP43F	**TGAGGTGGCAGAGGGCAGGT**TGATAGGTGGTATGTTTTCGCTTGAAC
pP43R	**AGTTCTTCTCCTTTACTCAT**GTGTACATTCCTCTCTTACCTATAATGG

^a^The underlined bold characters indicate the flanking sequence homologous to their respective template plasmid

^b^The underlined italic characters denote the inserted *Pst*I restriction site

**Table 3:** The sequences of constructed theophylline riboswitches

Theophylline riboswitches	Sequences^a^
riboE1	*ATACGACTCACTATA* ***GGTGATACCAGCATCGTCTTGATGCCCTTGGCAGCACC***CTGCTAAAGGAGGTAACAACAAG
riboE1-15	*ATACGACTCACTATA* ***GGTGATACCAGCATCGTCTTGATGCCCTTGGCAGCACC***CTGCTAAAGGAGGTAACAACAAGCTGCAG ^b^

^a^Italic constant sequences; bolditalic theophylline riboswitch

^b^Underline inserted *Pst*I site

### GFP expression by an engineered theophylline riboswitch E combined with P43

A single clone of *B. subtilis* 168 harbouring recombinant plasmids was plated on agar plates and individual colonies were inoculated into the test tube containing LB medium and cultured overnight at 37 °C with 200-rpm shaking. Then, the fresh culture was inoculated into 250-mL conical flasks containing 30-mL LB media and kanamycin (50 μg/mL), the initial OD_600_ was adjusted to 0.05, and the 250-mL flasks were incubated at 37 °C in the shaker at 200 rpm. To monitor the cell growth and reporter gene activation, cultures of *B. subtilis* harbouring pP43-*gfp* and pBSG11 were collected periodically before and after activation with 4 mM theophylline. To characterize the induction of P43′-riboE1, recombinant *B. subtilis* BSG11 harbouring pBSG11 were treated with 4 mM theophylline after 7 h in culture. Non-induced BSG11 was used as control to determine the expression level of leakage. Afterwards, the cultures were sampled periodically. To characterize the dose-dependency of BSG11, different concentrations of theophylline (1, 2, 4 or 8 mM) were added to the medium and the cultures were incubated for 24 h. Then, the recombinant bacterial cells were collected and the relative fluorescence intensities were measured with a microplate reader. To compare the expression levels from pBSG11 and pBSG10, the corresponding recombinant strains were treated with 8 mM theophylline for 12 and 24 h. Cultures treated with 4% DMSO were designated as controls.

### Reporter gene assay

The fluorescence measurements were conducted in accordance with a previously reported procedure, with some modifications [20]. In brief, the cultured recombinant *B. subtilis* cells were collected by centrifugation at 4000 rpm for 5 min and then the supernatant was discarded. The cell pellets were washed with potassium phosphate buffer (PBS, 137 mM NaCl, 2.7 mM KCl, 8 mM Na_2_PO_4_ 2H_2_O, 1.76 mM KH_2_PO_4_, adjust to pH 7.4 with 1 N HCl) three times. Then, the cell pellets were re-suspended in PBS with appropriate dilution, after which 200-μL aliquot of each sample was transferred to the wells of a black-walled transparent bottomed 96-well plate. The fluorescence intensity (a.u) and cell density (OD_600_) were simultaneously detected using an Infinite M200 microplate reader (Tecan Group Ltd, Männedorf, Switzerland), with excitation at 495/9 and emission at 535/9. Relative fluorescence units (a.u/OD_600_) were obtained by dividing the measured fluorescence intensity by the corresponding OD_600_ value, and were used to represent the level of GFP expression.

### SDS-PAGE analysis

A volume of 1 mL of the recombinant *B. subtilis* cells were harvested by centrifugation at 5000 rpm for 3 min. Then, equal volumes of PBS were added into the 1.5-mL tube and vortexed for 30 s prior to discarding the supernatant after centrifugation again under the same setting. Subsequently, the pellet was thoroughly suspended in 200-μL lysis buffer (100 mM PBS with 5 μg/mL lysozyme), then incubated at 37 °C for 10–15 min. Afterwards, the cells were destroyed completely by ultra-sonication. Then, the lysates were subjected to centrifugation at 14,000 rpm and 4 °C for 25 min. Then, 120-μL supernatant was pipetted and mixed with 30-μL 5× loading buffer. After the determination of the concentration of the extracted proteins by the Bradford method, equal amounts of total proteins were loaded onto the SDS-PAGE to analyse the expression levels.

### β-glucuronidase (GUS) enzymatic activity assay

To test the stability and compatibility of expression from the constructs, recombinant *B. subtilis* BSGgus was used for the determination of the expression level and enzymatic activity of GUS. BSGgus was cultured as described above for the GFP assays. The cultures were sampled periodically after induction by 8 mM theophylline. Samples were washed with PBS buffer three times and then lysed by the addition of equal volumes of lysis buffer at 37 °C for 30 min. After centrifugation at 4 °C for 20 min, the supernatants were collected and diluted to the appropriate concentration for the enzymatic activity assay. For quantification of the product, the standard curve was firstly plotted by measuring the absorbance of a concentration gradient (1, 2, 3, 4, 5, 6, 7 and 8 μg) of *p*-nitrophenol (Sigma-Aldrich, Shanghai, China) at 405 nm. Afterwards, the reactions were carried out in a 96-well plate and each reaction contained the following components: 50 μL of sample; 50 μL of the substrate 4-nitrophenyl β-d-glucuronide (PNPG, 0.5 mg/mL, Sigma-Aldrich, Shanghai, China). The reactions were incubated at 37 °C for 5 min, followed by the addition of 100 μL Na_2_CO_3_ (1 M) to stop the reaction. Then, the absorbance was measured at 405 nm using an Infinite M200 microplate reader. One unit is defined as the amount of enzyme that catalyses the production of 1 μmol of *p*-nitrophenol within 1 min. The enzymatic assay was independently performed in triplicates and the data are presented as the mean ± SD.

## Abbreviations

SD: Shine–Dalgarno
riboE1: riboswitch E1
P_srfA_: *srfA* promoter
P_aprE_: *aprE* promoter
P_aprE’_: *aprE* promoter lacking native SD sequence
P43′: P43 promoter lacking native SD sequence
IPTG: isopropyl β-d-1-thiogalactopyranoside
LB: Luria–Bertani medium
GUS: β-Glucuronidase
GFP: green fluorescent protein
DMSO: dimethyl sulfoxide
PNPG: β-d-glucuronide
*bla*: β-Lactamases
*Km*^r^: kanamycin resistance gene
